# Author Correction: Emergent increase in coral thermal tolerance reduces mass bleaching under climate change

**DOI:** 10.1038/s41467-023-44537-9

**Published:** 2024-01-04

**Authors:** Liam Lachs, Simon D. Donner, Peter J. Mumby, John C. Bythell, Adriana Humanes, Holly K. East, James R. Guest

**Affiliations:** 1 School of Natural and Environmental Sciences, Newcastle University, Newcastle upon Tyne, UK; 2 Institute of Resources, Environment and Sustainability, and Department of Geography, University of British Columbia, Vancouver, BC Canada; 3 Marine Spatial Ecology Lab, School of Biological Sciences, The University of Queensland, St Lucia, QLD Australia; 4 Palau International Coral Reef Center, Koror, Palau; 5 Department of Geography and Environmental Sciences, Northumbria University, Newcastle upon Tyne, UK

**Keywords:** Climate-change ecology, Evolutionary ecology, Climate-change impacts, Projection and prediction, Marine biology

Correction to: *Nature Communications* 10.1038/s41467-023-40601-6, published online 22 August 2023

The original version of this Article contained an error in the panel heading for Figure 5(b), which incorrectly read “Middle-of-the-road (SSP2–3.0)”. The correct version replaces this panel heading with “Middle-of-the-road (SSP2–4.5)”. This has now been corrected in both the HTML and the PDF versions of the Article.

