# Supplementary material for: Emergent increase in coral thermal tolerance reduces mass bleaching under climate change
Source: Nat Commun. 2023 Aug 22;14:4939. doi: 10.1038/s41467-023-40601-6 (PMC10444816; doi:10.1038/s41467-023-40601-6)
Supplement: Supplementary file 1 — Supplementary Information [file 41467_2023_40601_MOESM1_ESM.pdf]

Emergent increase in coral thermal tolerance reduces mass bleaching under climate change

Liam Lachs[1,2,*], Simon D. Donner[2], Peter J. Mumby[3,4], John C. Bythell[1], Adriana Humanes[1], Holly K. East[5], James R. Guest[1]

[1]School of Natural and Environmental Sciences, Newcastle University, Newcastle upon Tyne, United Kingdom

[2]Institute of Resources, Environment and Sustainability, and Department of Geography, University of British Columbia, Vancouver, British Columbia, Canada

[3]Marine Spatial Ecology Lab, School of Biological Sciences, The University of Queensland, St Lucia, Queensland, Australia

[4]Palau International Coral Reef Center, Koror, Palau

[5]Department of Geography and Environmental Sciences, Northumbria University, Newcastle upon Tyne, United Kingdom

[*]Correspondence to Liam Lachs, liamlachs@gmail.com

Supplementary Information

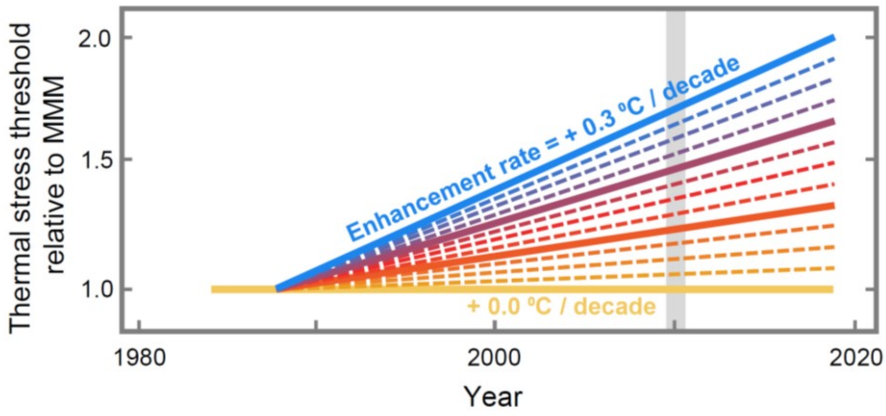

Supplementary Fig. 1 | Simulated increases in coral thermal tolerance. The operational temperature stress threshold (MMM + 1 °C, Maximum of Monthly Means, yellow line) was increased linearly at 13 different rates between 0.0 and 0.3 °C/decade, starting from 1988 (centre year of climatological baseline period). Then, timeseries of accumulated bleaching heat stress (Degree heating weeks – DHW) were calculated under each simulation from satellite-based sea surface temperature data, resulting in contrasting DHW profiles among simulated rates of thermal tolerance increase. A shaded grey region is show for the year 2010 (inspected in more detail in Fig. 2), showing the levels that thermal tolerance (temperature stress thresholds) would have reached after 22 years of simulated enhancement.

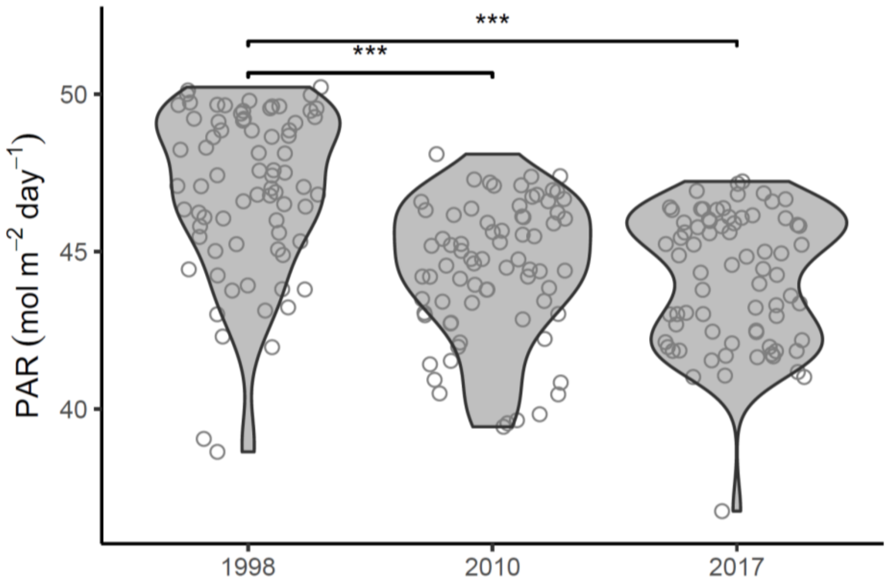

Supplementary Fig. 2 | Light regime for major heatwave years. Violin plots showing density kernels of average photosynthetically available radiation (PAR) during the heatwave period August to September for all Palauan reef pixels (points) between the main heatwave years. Statistical differences among groups were tested using a linear mixed effects model with random intercepts for latitude and longitude to account for spatial autocorrelation and linked to post-hoc Tukey test for pairwise comparisons. P value significance is given as asterisks for $P < 0.05$ (*), $P < 0.01$ (**), $P < 0.001$ (***).

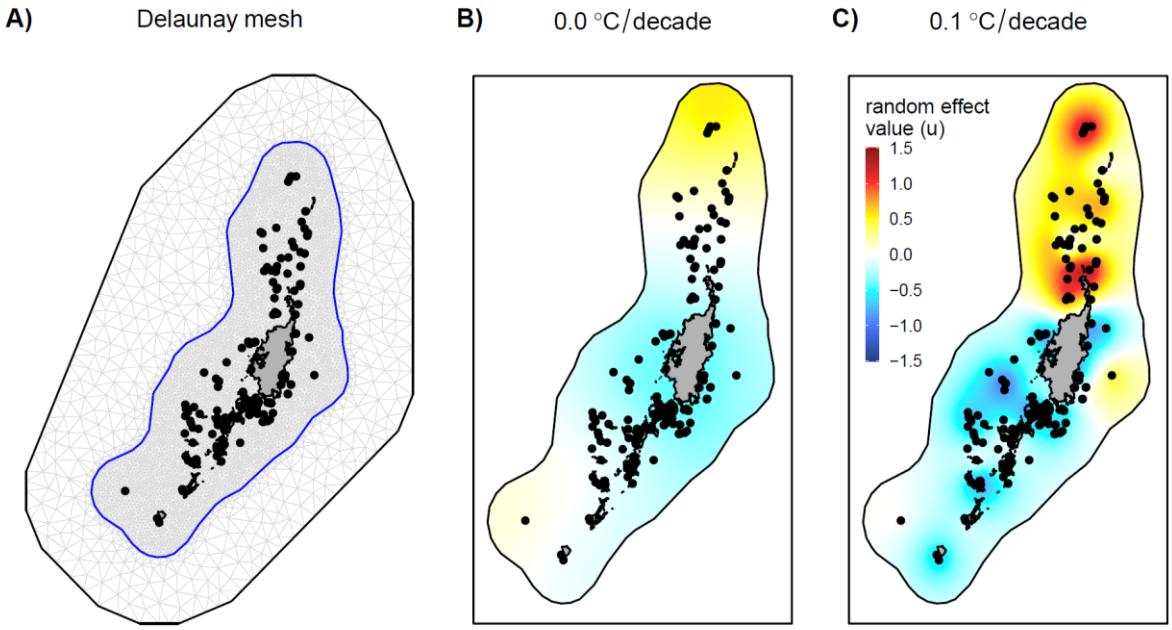

Supplementary Fig. 3 | Spatial autocorrelation. (A) Delaunay triangulation mesh on which the spatially correlated uncertainty was computed for prediction of bleaching observations based on DHW. (B, C) Examples of the spatial random fields for two bleaching prediction models, with a temporally fixed thermal tolerance set to 0.0 °C/decade (B) and with thermal tolerance enhancement set to increase at 0.1 °C/decade (C). Random effect values are shown under beta transformation.

Supplementary Table 1 | Summary of Global Circulation Models (GCM) data used. Coupled Model Intercomparison Project Phase 6 (CMIP6) GCM datasets used in this study for statistical downscaling of Sea Surface Temperature (SST) and future projection of mass coral bleaching conditions. From each GCM, SST were downloaded from historical runs and from future projections among different Shared Socioeconomic Pathway scenarios (SSP1, SSP2, SSP3, SSP5), each reflecting a different level of radiative forcing (2.6, 4.5, 7.0, 8.5 W m^{-2}, respectively). Source numbering corresponds to Supplementary Table 2.

Institution	GCM	Historical	SSP 1	SSP 2	SSP 3	SSP 5	Source
CSIRO	ACCESS-CM2	y	y	y	y	y	1
CSIRO	ACCESS-ESM1-5	y	y	y	y	y	2
BCC	BCC-CSM2-MR	y	y	y	y	y	3
CCCma	CanESM5	y	y	y	y	y	4
NCAR	CESM2	y	y	y	y	y	5
CNRM-CERFACS	CNRM-CM6-1	y	y	y	y	y	6
CNRM-CERFACS	CNRM-ESM2-1	y	y	y	y	y	7
EC-Earth-Consortium	EC-Earth3	y	y	y	y	y	8
EC-Earth-Consortium	EC-Earth3-Veg	y	y	y	y	y	9
MOHC	HadGEM3-GC31-LL	y	y	y	y	y	10
IPSL	IPSL-CM6A-LR	y	y	y	y	y	11
MIROC	MIROC6	y	y	y	y	y	12
MRI	MPI-ESM1-2-LR	y	y	y	y	y	13
MPI-M	MRI-ESM2-0	y	y	y	y	y	14
NUIST	NESM3	y	y	y	-	y	15
NCC	NorESM2-LM	y	y	y	y	y	16
MOHC	UKESM1-0-LL	y	y	y	y	y	17

Supplementary Table 2 | Global circulation model (GCM) data bibliography. References for each Coupled Model Intercomparison Project Phase 6 (CMIP6) GCM datasets used in this study for statistical downscaling of Sea Surface Temperature (SST) and future projection of mass coral bleaching conditions (*c.f.*, Extended Data Table 1).

No.	Reference
1	Dix M. *et al*. CSIRO-ARCCSS ACCESS-CM2 model output prepared for CMIP6 ScenarioMIP ssp585. (2019) doi:10.22033/ESGF/CMIP6.4332.
2	Ziehn T. *et al*. CSIRO ACCESS-ESM1.5 model output prepared for CMIP6 ScenarioMIP ssp585. (2019) doi:10.22033/ESGF/CMIP6.4333.
3	Xin X. *et al*. BCC BCC-CSM2MR model output prepared for CMIP6 ScenarioMIP ssp585. (2019) doi:10.22033/ESGF/CMIP6.3050.
4	Swart N. C. *et al*. CCCma CanESM5 model output prepared for CMIP6 ScenarioMIP ssp585. (2019) doi:10.22033/ESGF/CMIP6.3696.
5	Danabasoglu G. NCAR CESM2 model output prepared for CMIP6 CMIP. (2019) doi:10.22033/ESGF/CMIP6.2185.
6	Voldoire A. CNRM-CERFACS CNRM-CM6-1 model output prepared for CMIP6 ScenarioMIP ssp585. (2019) doi:10.22033/ESGF/CMIP6.4224.
7	Voldoire A. CNRM-CERFACS CNRM-ESM2-1 model output prepared for CMIP6 ScenarioMIP ssp585. (2019) doi:10.22033/ESGF/CMIP6.4226.
8	(EC-Earth) E.-E. C. EC-Earth-Consortium EC-Earth3 model output prepared for CMIP6 ScenarioMIP ssp585. (2019) doi:10.22033/ESGF/CMIP6.4912.
9	(EC-Earth) E.-E. C. EC-Earth-Consortium EC-Earth3-Veg model output prepared for CMIP6 ScenarioMIP ssp585. (2019) doi:10.22033/ESGF/CMIP6.4914.
10	Good P. MOHC HadGEM3-GC31-LL model output prepared for CMIP6 ScenarioMIP ssp585. (2020) doi:10.22033/ESGF/CMIP6.10901.
11	Boucher O. *et al*. IPSL IPSL-CM6A-LR model output prepared for CMIP6 ScenarioMIP ssp585. (2019) doi:10.22033/ESGF/CMIP6.5271.
12	Shiogama H. Abe M. & Tatebe H. MIROC MIROC6 model output prepared for CMIP6 ScenarioMIP ssp585. (2019) doi:10.22033/ESGF/CMIP6.5771.
13	Wieners K.-H. *et al*. MPI-M MPI-ESM1.2-LR model output prepared for CMIP6 ScenarioMIP ssp585. (2019) doi:10.22033/ESGF/CMIP6.6705.
14	Yukimoto S. *et al*. MRI MRI-ESM2.0 model output prepared for CMIP6 ScenarioMIP ssp585. (2019) doi:10.22033/ESGF/CMIP6.6929.
15	Cao J. NUIST NESMv3 model output prepared for CMIP6 ScenarioMIP ssp585. (2019) doi:10.22033/ESGF/CMIP6.8790.
16	Seland Ø. *et al*. NCC NorESM2-LM model output prepared for CMIP6 ScenarioMIP ssp585. (2019) doi:10.22033/ESGF/CMIP6.8319.
17	Good P. *et al*. MOHC UKESM1.0-LL model output prepared for CMIP6 ScenarioMIP ssp585. (2019) doi:10.22033/ESGF/CMIP6.6405.
