# Supplementary material for: Emergent increase in coral thermal tolerance reduces mass bleaching under climate change
Source: Nat Commun. 2023 Aug 22;14:4939. doi: 10.1038/s41467-023-40601-6 (PMC10444816; doi:10.1038/s41467-023-40601-6)
Supplement: Supplementary file 3 — Reporting Summary [file 41467_2023_40601_MOESM3_ESM.pdf]

nature portfolio

Corresponding author(s): Liam Lachs

Last updated by author(s): Jul 18, 2023

Reporting Summary

Nature Portfolio wishes to improve the reproducibility of the work that we publish. This form provides structure for consistency and transparency in reporting. For further information on Nature Portfolio policies, see our Editorial Policies and the Editorial Policy Checklist.

Statistics

For all statistical analyses, confirm that the following items are present in the figure legend, table legend, main text, or Methods section.

n/a	Confirmed	
☐	☒	The exact sample size (*n*) for each experimental group/condition, given as a discrete number and unit of measurement
☐	☒	A statement on whether measurements were taken from distinct samples or whether the same sample was measured repeatedly
☐	☒	The statistical test(s) used AND whether they are one- or two-sided<br>*Only common tests should be described solely by name; describe more complex techniques in the Methods section.*
☐	☒	A description of all covariates tested
☐	☒	A description of any assumptions or corrections, such as tests of normality and adjustment for multiple comparisons
☐	☒	A full description of the statistical parameters including central tendency (e.g. means) or other basic estimates (e.g. regression coefficient) AND variation (e.g. standard deviation) or associated estimates of uncertainty (e.g. confidence intervals)
☐	☒	For null hypothesis testing, the test statistic (e.g. *F*, *t*, *r*) with confidence intervals, effect sizes, degrees of freedom and *P* value noted<br>*Give P values as exact values whenever suitable.*
☐	☒	For Bayesian analysis, information on the choice of priors and Markov chain Monte Carlo settings
☐	☒	For hierarchical and complex designs, identification of the appropriate level for tests and full reporting of outcomes
☒	☐	Estimates of effect sizes (e.g. Cohen's *d*, Pearson's *r*), indicating how they were calculated

Our web collection on statistics for biologists contains articles on many of the points above.

Software and code

Policy information about availability of computer code

Data collection	Satellite-sensed Sea Surface Temperature (SST) datasets were accessed from NOAA Coral Reef Watch (https://coralreefwatch.noaa.gov/product/5km/index_5km_sst.php) using FileZilla version 3.48.0. Future projected datasets were accessed from the World Climate Research Programme Earth (WCRP) Coupled Model Intercomparison Project (CMIP6) (https://esgf-node.llnl.gov/search/cmip6/) using GNU Bash version 5.0.16(1).
Data analysis	All original R code (version 4.0.2) used for data analysis is available at https://doi.org/10.25405/data.ncl.21982484. Contained in the repository is sufficient data and code to reproduce all analyses in the study, however, any further guidance or additional information required is available from the lead contact upon request (e.g., downloading software/packages etc.).

For manuscripts utilizing custom algorithms or software that are central to the research but not yet described in published literature, software must be made available to editors and reviewers. We strongly encourage code deposition in a community repository (e.g. GitHub). See the Nature Portfolio guidelines for submitting code & software for further information.

Data

Policy information about availability of data

All manuscripts must include a data availability statement. This statement should provide the following information, where applicable:

- Accession codes, unique identifiers, or web links for publicly available datasets
- A description of any restrictions on data availability
- For clinical datasets or third party data, please ensure that the statement adheres to our policy

All original data has been deposited at https://doi.org/10.25405/data.ncl.21982484. The land mask for maps in this study were based on the NOAA National Centre for Coastal Ocean Science Data Collection (https://products.coastalscience.noaa.gov/collections/benthic/e102palau/), and the coral reef mask used was from the United Nations global distribution of coral reefs (https://doi.org/10.34892/t2wk-5t34). All datasets analysed are publicly available as of the date of publication.

Human research participants

Policy information about studies involving human research participants and Sex and Gender in Research.

Reporting on sex and gender	N/A
Population characteristics	N/A
Recruitment	N/A
Ethics oversight	N/A

Note that full information on the approval of the study protocol must also be provided in the manuscript.

Field-specific reporting

Please select the one below that is the best fit for your research. If you are not sure, read the appropriate sections before making your selection.

☐ Life sciences ☐ Behavioural & social sciences ☒ Ecological, evolutionary & environmental sciences

For a reference copy of the document with all sections, see nature.com/documents/nr-reporting-summary-flat.pdf

Ecological, evolutionary & environmental sciences study design

All studies must disclose on these points even when the disclosure is negative.

Study description	To test whether coral thermal tolerance enhancement has already occurred across Palau over the last four decades, we compiled a historic dataset of mass coral bleaching observations (N=240) and 36 years of daily satellite-based sea surface temperature data. We applied spatial Bayesian statistical modelling techniques to test the susceptibility of corals to accumulated heat stress (DHW) (models in the form Bleaching ~ B0 + B1*DHW + spatial_error + error). We then contextualise the different simulated rates of thermal tolerance enhancement under climate change, testing their influence on future bleaching trajectories based on SST data from an ensemble of statistically downscaled Global Circulation Models (GCMs) by tracking the frequency of bleaching conditions per decade (on a rolling basis) over the coming century.
Research sample	This study was conducted with Palau as total domain, and point bleaching observations as the sampling unit given that is the only available historic data. It was based on a novel analysis of 3 datasets: historic satellite-sensed sea surface temperatures (https://coralreefwatch.noaa.gov/product/5km/index_5km_sst.php), future projected sea surface temperatures from GCMs (https://esgf-node.llnl.gov/search/cmip6/), and mass coral bleaching survey observations (https://doi.org/10.5281/zenodo.6780843)
Sampling strategy	The sample size for bleaching observations was based on the extent of historic surveys (N = 237 point observations of bleaching severity). This is a subset of the global dataset provided by Virgen-Urselay (2023). Such a sample size is sufficient to run the spatial regression models used in this study (e.g., only two fixed effect parameters to be estimated, an intercept and a slope). A 'sampling strategy' and determination of is not applicable to the thermal datasets as these were simply used to estimate the thermal regime associated with each record of bleaching severity.
Data collection	Data contributors to the subset of the global bleaching dataset which was used for this study can be found in the associated dataset (https://doi.org/10.5281/zenodo.6780843). As described in Virgen-Urcelay et al., 2023 (https://doi.org/10.1371/journal.pone.0281719), to account for multiple survey methods in the data collection (e.g., photo transects, point intercept transects, video transects), bleaching observations in the database (reported as percentage of corals bleached) were summarised as severity scores ranging from 0 to 3: 0 = no bleaching (0%), 1 = mild bleaching (1-10%), 2 = moderate bleaching (11-50%), and 3 = severe bleaching (>50%).
Timing and spatial scale	Bleaching data were available between 06/1998 and 07/2017. Daily 5km satellite-based sea surface temperature data was used from 01/1985 to 12/217. GCM-based daily sea surface temperatures were used between 01/1985 and 12/2100. The study was focused on

	the reefs of Palau which occurring in a domain of 0.5 degrees of longitude by 2 degrees of latitude
Data exclusions	No data were excluded from the analysis, since all bleaching survey observations were from the warm season of the year when heat stress-induced bleaching may be expected.
Reproducibility	Data and code are provided on a public repository to allow full reproducibility of this study.
Randomization	This is not applicable to this modeling study, as no field or lab experiments were conducted. However, to account for spatial autocorrelation in bleaching prediction models based on DHW explicitly, we used Integrated Nested Laplace Approximation (INLA) combined with a spatial Matérn correlation.
Blinding	This is not applicable, given that this modeling study did not generate any new experimental or observational datasets.

Did the study involve field work? ☐ Yes ☒ No

Reporting for specific materials, systems and methods

We require information from authors about some types of materials, experimental systems and methods used in many studies. Here, indicate whether each material, system or method listed is relevant to your study. If you are not sure if a list item applies to your research, read the appropriate section before selecting a response.

Materials & experimental systems

n/a	Involved in the study	
☒	☐	Antibodies
☒	☐	Eukaryotic cell lines
☒	☐	Palaeontology and archaeology
☒	☐	Animals and other organisms
☒	☐	Clinical data
☒	☐	Dual use research of concern

Methods

n/a	Involved in the study	
☒	☐	ChIP-seq
☒	☐	Flow cytometry
☒	☐	MRI-based neuroimaging
